# Human Apoptotic Cells, Generated by Extracorporeal Photopheresis, Modulate Allogeneic Immune Response

**DOI:** 10.3389/fimmu.2019.02908

**Published:** 2019-12-18

**Authors:** Caroline Pilon, Thomas Stehlé, Asma Beldi-Ferchiou, Marie Matignon, Allan Thiolat, Aude Burlion, Cynthia Grondin, Brigitte Birebent, France Pirenne, Hélène Rouard, Philippe Lang, Gilles Marodon, Philippe Grimbert, José L. Cohen

**Affiliations:** ^1^Assistance Publique-Hôpitaux de Paris (AP-HP), Groupe Hospitalo-Universitaire Chenevier Mondor, Centre d'Investigation Clinique Biothérapie, Créteil, France; ^2^Institut Mondor de recherche biomédicale, Université Paris-Est, UMR_S955, UPEC, Créteil, France; ^3^Inserm, U955, Equipe 21, Créteil, France; ^4^AP-HP, Groupe Hospitalo-Universitaire Chenevier Mondor, Service de Néphrologie-Transplantation, Créteil, France; ^5^Sorbonne Universités, UPMC Univ Paris 06, INSERM, CNRS, Centre d'Immunologie et des Maladies Infectieuses (CIMI-Paris), Paris, France; ^6^Etablissement Français du Sang (EFS) – Ile de France, Créteil, France; ^7^Inserm, U955, Equipe 2, Créteil, France

**Keywords:** tolerance, NSG mice, immunomodulation, transplantation, apoptotic cell

## Abstract

The induction of specific and sustainable tolerance is a challenging issue in organ transplantation. The discovery of the immunosuppressive properties of apoptotic cells in animal models has paved the way for their use in human transplantation. In this work, we aimed to define a stable, reproducible, and clinically compatible production procedure of human apoptotic cells (Apo-cells). Using a clinically approved extracorporeal photopheresis technique, we have produced and characterized phenotypically and functionally human apoptotic cells. These Apo-cells have immunosuppressive properties proved *in vitro* and *in vivo* in NOD/SCID/γC mice by their capacity to modulate an allogeneic response following both a direct and an indirect antigen presentation. These results brought the rationale for the use of Apo-cells in tolerance induction protocol for organ transplantation.

## Introduction

Organ transplantation has become a successful therapeutic strategy for the treatment of many end-stage organ failures. It requires, however, long-term immunosuppressive treatment to overcome the allogeneic immune response, and that in turn is associated with toxicity and high incidence of malignancies, infections, and metabolic diseases. One of the ultimate goals for researchers is to develop novel transplantation therapeutic approaches that induce donor-specific tolerance. By controlling allogeneic recognition, responsible for acute and chronic allograft rejection, it would be possible to lessen the generalized immunosuppression and its associated side effects.

Extracorporeal photopheresis (ECP) appears as a promising strategy to achieve the desired goal. ECP is a therapeutic regimen approved as a palliative treatment for the skin manifestations associated with cutaneous T-cell lymphoma (1). In transplantation, ECP has also been proven effective in a variety of clinical indications including solid organ transplantation (2) and hematopoietic stem cell transplantation (3). ECP can be in some cases an effective approach in steroid-resistant chronic graft-vs.-host disease (GvHD), thus sparing patients the glucocorticoid or immunosuppressant therapies (4). Thanks to its efficacy and the few related adverse effects, ECP is highly recommended for the prevention of graft rejection in heart transplantation (5), and can be proposed as a prophylaxis for GvHD in myeloablative HLA-matched allogeneic bone marrow transplantation (6) and in grade I and II acute GvHD (3). On the same clinical setting, a recent study has also shown the interest of combining ECP with other immune-based approaches like low-dose IL-2 (7).

Despite its therapeutic interest and wide-range coverage, the mechanisms of action of ECP are poorly known and sometimes contradictory. Surprisingly, it was reported that ECP promoted the release of prototypic immune-stimulatory cytokine IL-1β (8) and that the treated monocytes retained their ability to differentiate into fully functional dendritic cell (DC) that maturated and stimulated T cells as well as normal DC (9). On the other hand, a bunch of arguments, built on data collected mainly on rodents, strongly suggest that ECP-induced immunomodulation could be mediated largely by apoptosis resulting from exposure of mononuclear cells to UV-A or UV-B. Apoptotic cells have the ability to down-regulate the inflammatory and the immune responses by delivering inhibitory signals to phagocytes (10, 11), particularly DCs (12). The latter internalize cells in early apoptosis; exhibit a selective decrease in the levels of pro-inflammatory cytokines IL-1a, IL-1b, IL-6, IL-12p70, and TNF-α; and secret normal or increased amounts of immunosuppressive TGF-β and IL-10 (13–15). Given the efficient capacity of DC to internalize apoptotic cells (16) making it a key player in peripheral T-cell tolerance (17), direct administration of donor apoptotic cells could be envisaged to simultaneously deliver both inhibitory signals and donor allogeneic antigens to recipient DC. For such, a study has shown that intravenous administration of donor apoptotic cells is an alternative method to prevent DC activation and to present donor allogeneic antigen to recipient DC leading to a significant control of the anti-donor response in a rat model of heart transplantation (18). Such results open new perspectives for donor-specific tolerance induction in solid organ transplantation in human.

Although preclinical results are robust in animal models of transplantation, data on the characterization and immunosuppressive properties of apoptotic cells obtained from human samples remain rare. *In vitro*, it was suggested that 8-MOP-treated and UV-A-irradiated cells could acquire a partial immunogenic phenotype that triggers phagocytosis of apoptotic cells by macrophages and DC; however, it was not enough to induce DC maturation and T-cell activation (19). In the present study, we used apoptotic cells generated from human peripheral blood mononuclear cells (PBMCs) that were incubated with 8-MOP and exposed to UV-A radiation (referred to as Apo-cells); the procedure and the tools are clinically validated. After phenotypic characterization, induction of primary and secondary mixed lymphocyte reaction (MLR), and *in vivo* testing of allogeneic immune responses, we demonstrate for the first time the capacity of human Apo-cells, obtained by ECP, to down-regulate allogeneic T-cell response. This work opens new therapeutic perspectives for allograft management.

## Materials and Methods

### Blood Samples

Human PBMCs were obtained from unrelated and unmatched healthy donors (*Etablissement Francais du Sang, Créteil, France*). PBMCs were isolated by density gradient centrifugation (Lymphocyte Separation Medium; Eurobio®, France), and the cells were suspended in RPMI-1640 + GluMAX (Life Technologies, France) supplemented with penicillin/streptomycin (Life Technologies, France) and 10% fetal bovine serum (Life Technologies, France).

### Induction and Labeling of Apoptotic Cells

Apoptosis of PBMC was induced by UV-A irradiation after 8-MOP (Methoxsalene, Macopharma) sensitization, as follows: PBMCs were transferred to a bag specially adapted for UV-A irradiation (Macopharma, *Mouvaux*, France), and 200 ng/ml of 8-MOP was added for 15 min at 37°C before UV-A irradiation at 2–4 J/cm^2^ (*Vilbert-Lourmat, Bio-Génic*, or *Macogenic*, Macopharma). Treated cells were then incubated at 37°C. After a 16 to 18 h incubation, UV-treated cells were incubated with 7AAD (BD Biosciences) for 15 min at room temperature, and then DiOC6 (67 μM) was added and cells were incubated again for 15 min at 37°C before flow cytometry analysis. DiOC6/7AAD (3,3-dihexyloxacarbocyanine Iodide/7-amino-actinomycinD) staining dyes were used to quantify apoptosis and necrosis. DiOC6 (Molecular Probes® Life Technologies™, ref D-273) was used to monitor potential mitochondrial trans-membrane disruption.

### Allogeneic MLR: Primary and Secondary

The responding cells (PBMCs) were first CFSE-labeled [5(6)-carboxyfluorescein diacetate N-succinimidyl ester; Sigma-Aldrich] and incubated at a ratio 1:1 (concentration of 1 × 10^6^ cells/ml) with stimulating allogeneic cells [Apo-cells or gamma-irradiated (20 Gy) PBMCs] at 37°C for 5 days. As a control, non-stimulated responding cells were cultivated in medium alone. The primary MLR was initiated by cultivating CD2– cells (CFSE-labeled), obtained from stimulating donor cells or non-labeled apoptotic cells, with allogeneic responding cells for 5 days. For the secondary stimulation, CD4-CD8 living cells from responding donor (CD4^+^, CD8^+^, CFSE^−^, 7AAD^−^) were sorted by fluorescence-activated cell sorting (FACS) (MoFlo Legacy, Beckman Coulter), then stained with cell proliferation dye eFluor450 (eBiosciences, France), and cultured with initial PBMCs from stimulating donor or third-party PBMCs at a ratio 1:1.

### Autologous MLR

Antigen-presenting cells (APCs) (CD2–) were first separated from T cells and NK cells by CD2 isolation. CD2+ was obtained by magnetic selection using Miltenyi technology (*Miltenyi Biotec*). PBMCs were first incubated with corresponding microbeads (anti-CD2) for 15 min and enriched in desired cells upon passage through large magnetic selection columns, according to the manufacturer's instructions. The obtained APCs (CD2–) were cultivated with allogeneic Apo-cells or control PBMCs at a ratio 1:1 for 48 h. Phenotypic analysis was then performed. For autologous MLR, the 48 h treated APCs were washed and then cultured with their own, previously isolated, CFSE-labeled CD2+ cells and kept at 37°C for 48 h at a ratio 1:1 for another 5 days.

### Cytokine Production

The amount of human IFNγ, IL-6, and TNF-α in the culture supernatant was determined using Duoset ELISA (R&D Systems) according to the manufacturer's instruction.

### mRNA Isolation and Real-Time Quantitative PCR

mRNA was extracted from 5 × 10^6^ PBMCs or UV-A-treated PBMCs using the RNeasy mini kit (Qiagen, Valencia, CA) according to the manufacturer's instructions. Genomic DNA was removed by DNase treatment (Qiagen). Total RNA was reverse transcribed to complementary DNA using reverse transcription reagents (Thermo Scientific, *Courtaboeuf* , *France*). Real-time quantitative PCR was performed using commercially available primer and probe sets (Applied Biosystems): HPRT, Hs99999909_m1; BAX, Hs00180269_m1; BCL2, Hs00608023; CASP3, Hs00234387; IL-10, Hs00961622_m1; and TGF-β1, Hs00998133_m1. We used the 2–ΔΔct method to calculate the relative expression of RNAs between a sample and a reference. All samples were tested in duplicates in 96-well plates with 7900HT fast real-time PCR system (Applied Biosystems, Foster City, CA, USA). HPRT was used as endogenous controls to normalize RNA amounts.

### Flow Cytometry

The antibodies used for FACS analysis are summarized in Table 1. The efluor 450-labeled anti-Foxp3 staining was performed using the eBioscience kit and protocol. The fixation/permeabilization kit (BD Biosciences) was used for intra-cellular staining of active caspase-3. Cell proliferation dye 450 (ebioscience) was used to help exclude APCs in the *in vivo* experiment. Flow cytometry events were acquired on a FACSCanto II flow cytometer (BD Biosciences) and analyzed using FlowJo (Tree Star, Ashland, OR, USA) software.

**Table 1:** Antibodies used for flow cytometry experiments and *in vitro* assay.

	**Fluorochrome**	**Clone**	**Supplier**		**Fluorochrome**	**Clone**	**Supplier**
CD3	PE	UCHT1	BD biosciences	CD45RO	PerCP	UCHL1	Miltenyi
CD3	V450	UCHT1	BD biosciences	CD56	APC	B159	BD biosciences
CD4	APC	RPA-T4	BD biosciences	CD69	APC-Cy7	FN50	BD biosciences
CD4	PE	RPA-T4	BD biosciences	CD86	APC	2331(FUN-1)	BD biosciences
CD4	APC-Cy7	RPA-T4	BD biosciences	CD95	FITC	DX2	BD biosciences
CD8	FITC	RPA-T8	BD biosciences	CD127	PerCP-Cy5.5	HIL-7R-M21	BD biosciences
CD8	PE	RPA-T8	BD biosciences	CCR7	PE-Cy7	3D12	BD biosciences
CD8	PE-Cy7	RPA-T8	BD biosciences	Foxp3	eFluor450	PCH101	eBiosciences
CD11c	PE	B-Ly6	BD biosciences	HLA-DR	V500	G46-6	BD biosciences
CD14	PE-Cy7	M-A251	BD biosciences	ICOS	PE	DX29	BD biosciences
CD19	V500	HIB19	BD biosciences	OX40	APC	ACT35	Miltenyi
CD25	PE-Cy7	2A3	BD biosciences	TGFβ	Purified	1D11.16.8	Ebiosciences
Active Caspase-3 Caspase-3	PE	C92-6095	BD biosciences				

### *In vivo* Experiment

Ten- to fourteen-week-old NOD/SCID/γC (NSG) mice, bred in our animal facility under specific pathogen-free conditions, were used for the purpose. All experimental protocols were approved by the local ethical committee (Ce5/2012/025) and are in compliance with the European Union guidelines. For the first infusion, 5 × 10^6^ Apo-cells or 5 × 10^6^ CD2– cells (cell proliferation dye-labeled to separate them by flow cytometry) were co-injected intravenously with 5 × 10^6^ allogeneic CD2+ cells. At day 6, 0.5 × 10^6^ CD2– cells (cell proliferation dye-labeled) from the first donor were injected. 5-Ethynyl-2′-deoxyuridine (EdU) was injected (intraperitoneally) at days 10 and 11. At day 11 (2 h after EdU second injection), the mice were sacrificed and their spleens harvested. Percentages of EdU+ cells were assessed using Click-iT™ EdU Alexa Fluor™ 488 Flow Cytometry Assay Kit (ThermoFisher) according to the manufacturer's protocol.

### Statistical Analysis

Results are expressed as mean ± SEM for flow cytometry and ELISA analysis or as median [interquartile] for quantitative PCR. We used paired or unpaired Student *t*-test or one-way ANOVA with *post-hoc* analysis depending on the number of comparatives to calculate *p*-values with Prism 5.0 (Graph Pad Software, Inc., La Jolla, CA, USA). The means were considered significantly different (rejection of the null hypothesis) if *p* < 0.05. No *a priori* statistical testing was performed to check the adequacy of the number of samples with statistical power: ^*^p < 0.05, ^**^p < 0.01, ^***^p < 0.001, ^****^p < 0.0001.

## Results

### Exposure of Human PBMCs to UV-A Induces Apoptosis and Phenotypic Modifications

**Figure 1:**
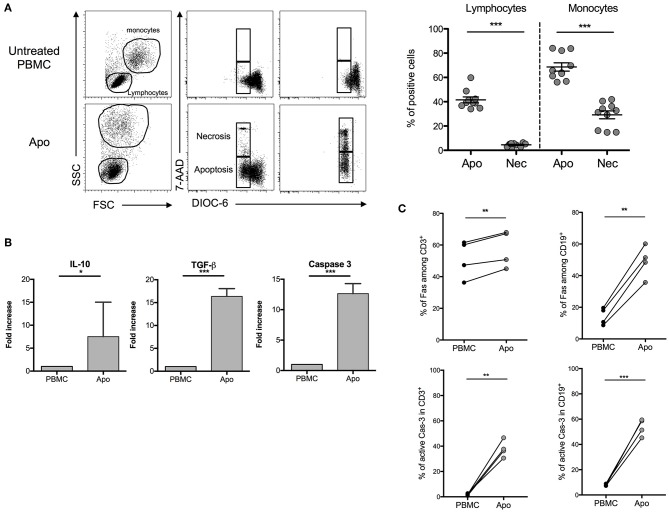
Characterization of apoptotic cells after UV-A treatment. Human peripheral blood mononuclear cells (PBMCs) were UV-A irradiated (2–4 J/cm^2^) after incubation with 8-MOP for 15 min at 37°C. Apoptosis was assessed 16 h after irradiation by DiOC6/7AAD staining (DiOC6^−^/7AAD^−^ = apoptotic cells, DiOC6^−^/7AAD^+^ = necrotic cells). **(A)** On the left, FSC-SSC modification and 7AAD/DiOC6 staining are shown for one experiment. On the right, the graphs show cumulative individual and mean ± SEM (horizontal bars) data from 10 independent donors. Statistics: unpaired Student *t*-test (***p < 0.001). **(B)** Real-time quantitative PCR was done on apoptotic cells after 16 h of post-irradiation incubation. mRNA fold increase was normalized on untreated PBMCs from the same donor. Caspase-3, TGF-β, and IL-10 expressions are shown (median [interquartile range], *n* = 7 donors). Statistics: paired Student *t*-test (***p < 0.001, *p < 0.05). **(C)** Fas and active caspase-3 (Cas-3) expressions by flow cytometry on PBMCs and UV-A-irradiated cells (Apo-cells) 16 h after irradiation in T and B cells (*n* = 4). Statistics: paired Student *t*-test (**p < 0.01).

In order to stay close to a cell product potentially available in humans, we used throughout this study a procedure based on exposing healthy donors' PBMCs to UV-A irradiation after 8-MOP sensitization, a procedure of ECP already in use in clinics. A first step was consisted of characterizing the cellular product and defining the optimal ECP procedure for the generation of strong apoptosis (potentially immunosuppressive) with reduced necrosis (potentially pro-inflammatory). After different incubation periods that followed for UV-A exposure, DiOC6/7AAD staining was performed to discriminate necrosis (7AAD^+^/DiOC6^−^) from apoptosis (7AAD^−^/DiOC6^−^). We observed that the larger cells, of mainly monocytes, are more sensitive to ECP than lymphocytes as attested by the forward scatter and side scatter parameters. The lower rates of necrotic cells in lymphocytes and monocytes (4.52 ± 0.47% and 29.19 ± 3.27%, respectively) and the higher rates of apoptotic cells in the same cell populations (41.52 ± 2.43% and 68.64 ± 3.44%, respectively) were obtained after 16 h of incubation (Figure 1A) as compared with shorter or longer periods of incubation (data not shown). Concomitantly, the levels of TGF-β and IL-10 mRNA were remarkably increased in ECP-treated cells (also called Apo-cells), suggesting a potential shift to an immunosuppressive profile generated by 8-MOP sensitization and UV-A exposure (Figure 1B). The increased expression level of caspase-3 mRNA (involved in early apoptotic cascade) observed in Apo-cells as compared with PBMCs suggests apoptosis induction after UV-A irradiation (Figure 1B), as well as Fas protein expression both in T and B cells (Figure 1C). Caspase-3 mRNA up-regulation has been reported as an indicator of TCR activation independently of caspase-3 activity and the induction of apoptosis (20). In our work, the percentage of active caspase-3 was also dramatically increased in both Apo-CD3 and Apo-CD19 cells (Figure 1C), which reinforces the notion of apoptosis triggered under these experimental conditions. Thus, we defined the “16 h of post-UV-A exposure incubation at 37°C” as the optimal procedure to generate Apo-cells.

**Figure 2:**
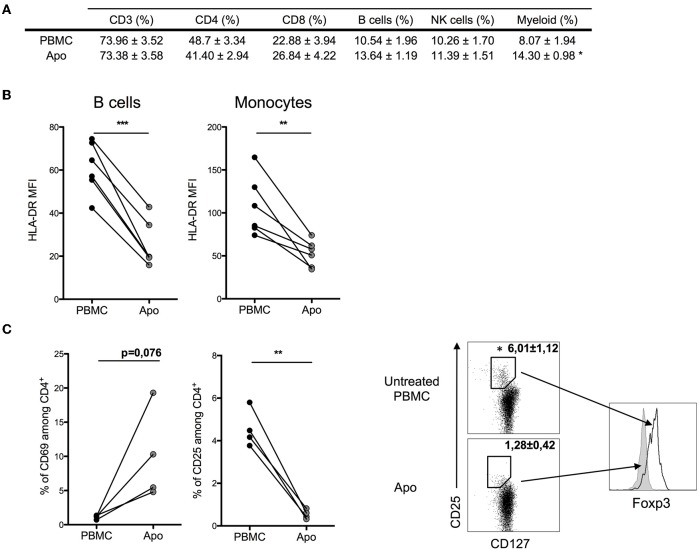
Effect of UV-A treatment on lymphocytes population. Untreated PBMCs and UV-A-irradiated cells (Apo-cells) from the same donor were phenotypically characterized after 16 h of incubation. **(A)** Lymphocytes populations were analyzed and surface-stained using CD3+ CD4+/CD8 for T cells, CD19+ for B cells, and CD56+ for NK cells and myeloid cells based on FSC-SSC size (mean ± SEM) *n* = 4–6 independent experiments. **(B)** HLA-DR mean fluorescence intensity (MFI) was assessed on B cells and monocytes (*n* = 6 independent experiments). Statistics: paired Student *t*-test (*p < 0.05, **p < 0.01, ***p < 0.001). **(C)** Activation markers CD25 and CD69 were analyzed on CD4 T cells; T-reg population was described by activation markers CD127^low^CD25^high^Foxp3^+^ (*n* = 3–4 independent experiments). Statistics: paired Student *t*-test (*p < 0.05, **p < 0.01).

When we looked at the relative proportions of T, NK, and B cells, we could not detect any substantial changes (Figure 2A). However, the expression of MHC class II molecules (HLA-DR) on monocytes and B-lymphocytes decreased in Apo-cells, suggesting decreased antigen presentation capacity in these cell populations (Figure 2B). CD4+ T cells in PBMCs did not express CD69, whereas we could detect 5–20% of CD4+CD69+ T cells in Apo-cells. In contrast, the subpopulation of CD4+CD25+ T cells dropped from 4–6% to a residual rate in Apo-cells in consistency with the disappearance of CD4+CD127–Foxp3+ subpopulation of T-reg (Figure 2C).

### Human Apoptotic Cells Induce Hypo-Responsiveness of Allogeneic Lymphocytes by Both Direct and Indirect Presentation Pathways

**Figure 3:**
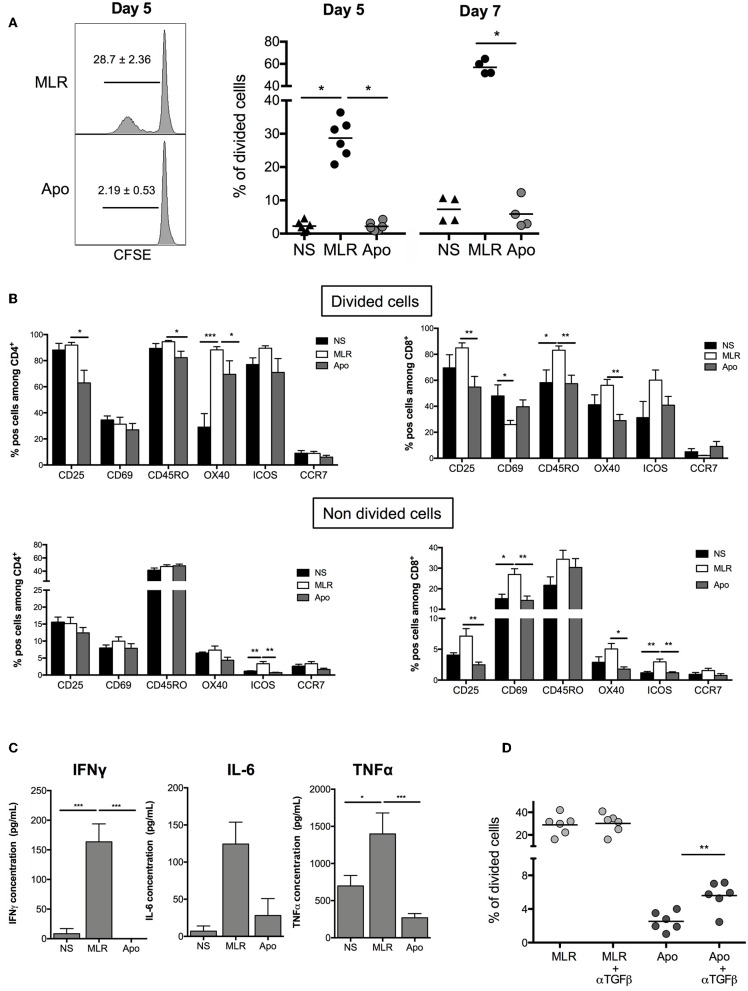
Apoptotic cells induce hyporesponsiveness of allogeneic cells: direct presentation pathway. Apo-cells and untreated PBMCs from the same donor were cultured with allogeneic cells (PBMCs). **(A)** CFSE responding cells were cultured for 5 or 7 days at a ratio 1:1 with either Apo-cells or PBMCs, and proliferation was analyzed by flow cytometry. On the left, one representative CFSE dilution from both conditions at day 5; on the right, the graph shows cumulative individual and mean ± SEM (horizontal bars) data from four to six independent experiments for day 5 and day 7. Statistics: one-way ANOVA with *post-hoc* (*p < 0.05). **(B)** Results are presented as mean ± SEM of the percentages of activation markers CD25^+^, CD69^+^, CD45RO^+^, OX40^+^, ICOS^+^, and CCR7^+^ cells on allostimulated T cells and on non-stimulated T cells (NS) at day 5 for divided cells (up) and non-divided cells (down) for CD4^+^ cells (left) and CD8^+^ cells (right). **(C)** Results are presented as mean ± SEM of supernatant concentrations of IFNγ, IL-6, and TNF-α cytokine analyzed by ELISA at day 5. Graphs show cumulative date of four to six independent experiments. Statistics: one-way ANOVA with *post-hoc* (*p < 0.05, **p < 0.01, ***p < 0.001). **(D)** CFSE responding cells were cultured for 5 days at a ratio of 1:1 with or without TGF-β neutralizing antibody, and proliferation was analyzed by flow cytometry. Percentages of divided cells after 5 days of co-culture. Graphs show cumulative individual data from six independent experiments. Statistics: unpaired Student *t*-test (**p < 0.01).

We wanted to establish the ability of Apo-cells to generate allogeneic response *in vitro*. In order to imitate the mechanism of direct alloantigen presentation, Apo-cells were incubated with allogeneic PBMCs. In the MLR groups, we used γ-irradiated cells to activate allogeneic PBMCs. In such a setting, γ-irradiation could also induce apoptosis. However, apoptosis/necrosis profile strongly differed notably for monocytes (Supplemental Figure 1) as compared with Apo-cells (Figure 1). After 5 days of co-culture, Apo-cells did not induce proliferation of allogeneic PBMCs as attested by CFSE dilution compared with standard MLR using untreated irradiated PBMCs from the same donor as stimulating cells (2.20 ± 0.53% vs. 28.68 ± 2.36%, respectively; Figure 3A). The low proliferation rate induced by Apo-cells does not seem to be related to the response time since the differences from irradiated PBMCs were even more pronounced when the culture was prolonged to day 7 (56.93 ± 3.17% for PBMCs vs. 5.88 ± 2.26% for Apo-cells). In addition to the decreased proliferation of responding cells in the presence of Apo-cells, T-cell activation markers (CD25, CD69, OX40, and ICOS) as well as memory phenotype markers (CD45RO and CCR7) were analyzed (Figure 3B). In the divided cells, we observed a statistically significant reduction in the proportion of CD4+ T cells expressing CD25 and in CD8+ T cells expressing CD25 and OX40, with a trend to a decrease in ICOS in Apo-cells as compared with irradiated PBMCs. The activated/memory marker CD45RO decreased in both CD4 and CD8, whereas there was no significant change in CCR7 expression. In the non-divided cells, significant changes of expression were observed mainly in CD8 population with a decrease in CD25, CD69, and OX40 and a trend to a decrease in ICOS expression in Apo-cells compared with irradiated PBMCs. In CD4 cells, only ICOS and CCR7 decreased (Figure 3B). The low priming capacity of Apo-cells was also reflected via the poor secretion of inflammatory cytokines such as IFNγ, IL-6, and TNF-α by responding cells, as compared with cells cultured with irradiated PBMCs (Figure 3C).

It was previously shown that apoptotic cells may exert their suppressive activity by secreting TGF-β (10). In our hands, the level of mRNA TGF-β was elevated in Apo-cells (Figure 1B). Interestingly, when we quantified TGF-β concentration in the culture at day 5, we did not observe any difference upon using irradiated PBMCs or Apo-cells (data not shown). By then, we reproduced MLR experiments in the presence of anti-TGF-β. In this setting, Apo-cell stimulatory capacity was partially restored (Figure 3D), suggesting that TGF-β produced in the presence of apoptotic cells might be partly responsible for the poor response of allogeneic responding cells. Blocking IL-10 had no effect on the culture (data not shown).

**Figure 4:**
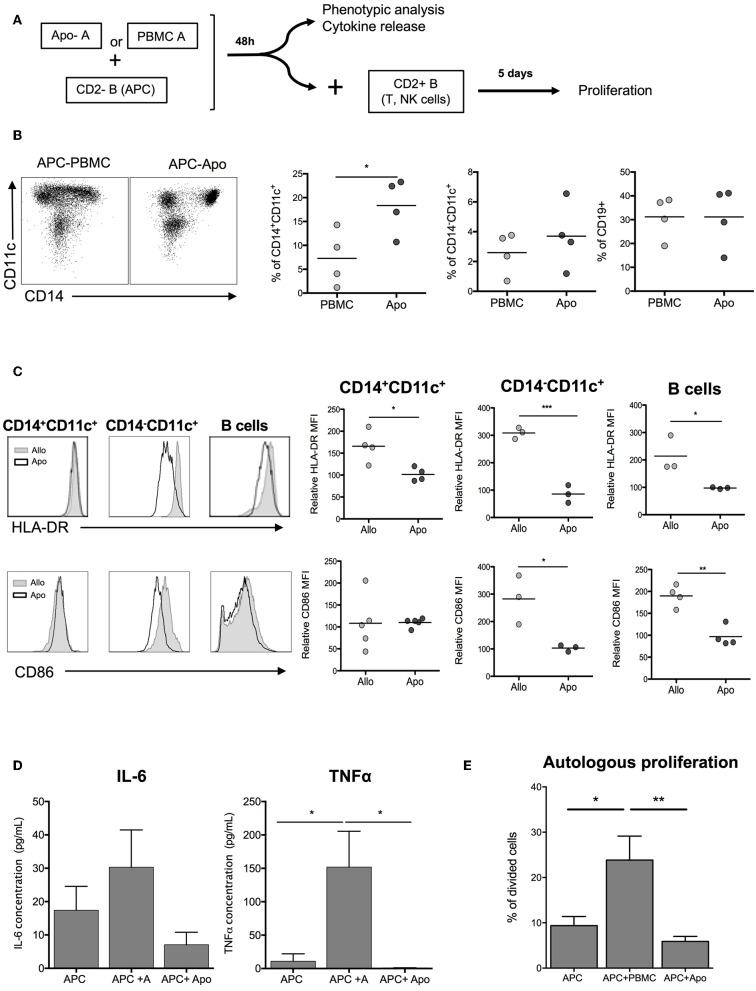
Apoptotic cells induce hyporesponse of allogeneic cells: indirect presentation pathway. **(A)** Experimental scheme. APCs (CD2– cells) were incubated with allogeneic Apo-cells or untreated irradiated-allogeneic PBMCs for 48 h. Phenotypic analysis was done on APCs, which were then used to stimulate autologous, CFSE-stained CD2+ cells. **(B)** Flow cytometry analysis of APCs showing the ratio of CD14+CD11c–, CD14+CD11c, and CD19+ cells. **(C)** Left: overlay comparison of HLA-DR and CD86 expression of allo and Apo-cells. Right: HLA-DR and CD86 relative MFI (normalized on the expression on APCs cultivated alone for 48 h) (*n* = 3–4 independent experiments). Statistics: unpaired Student *t*-test (*p < 0.05, **p < 0.01, ***p < 0.001). **(D)** Results are presented as mean ± SEM of supernatant concentration of IL-6 and TNF-α cytokine analyzed by ELISA after 48 h. Graphs show cumulative data of four independent experiments. Statistic: one-way ANOVA with *post-hoc* (*p < 0.05). **(E)** CFSE autologous responding cells were cultured for 5 days at a ratio 1:1 with APCs, APCs + PBMCs, or APCs + Apo-cells, and proliferation was analyzed by flow cytometry; cumulative individual and mean ± SEM (horizontal bars) data from five independent experiments. Statistics: 1-way ANOVA with *post-hoc* (*p < 0.05, **p < 0.01).

In order to evaluate the role of Apo-cells in the indirect presentation of alloantigens, we first incubated APCs (CD2– cells, containing mainly B cells and monocytes) with allogeneic Apo-cells or untreated irradiated allogeneic PBMCs from the same donor and studied the impact on the activation of APC (Figure 4A). After 48 h of co-culture of allogeneic Apo-cells with CD2– cells, the percentage of CD14+CD11c+ myeloid cells increased as compared with co-culture with untreated PBMCs (18.35 ± 2.9% vs. 7.28 ± 2.92%, respectively; Figure 4B). When we looked at HLA-class II expression on CD14+CD11c+, CD14–CD11c+, and CD19+ cells, in all the HLA-DR, mean fluorescence intensity was statistically decreased when CD2– cells were incubated with Apo-cells as compared with irradiated PBMCs (Figure 4C). CD86 is expressed on APC and known to provide costimulatory signals required for T-cell activation and survival. In the presence of allogeneic Apo-cells, the mean fluorescence intensity of CD86 expression on CD14–CD11c+ DCs and CD19 B cells was decreased compared with CD2– cells incubated with allogeneic PBMCs (Figure 4C). In alignment, co-incubation of CD2– with Apo-cells induced a dramatically reduced production of pro-inflammatory cytokines IL-6 and TNF-α compared with co-incubation with irradiated PBMCs (Figure 4D). The ability of pre-incubated CD2– cells to induce proliferation of autologous lymphocytes was analyzed. CD2– cells sorted out at day 0 were cultured from day 2 with CSFE-labeled autologous CD2+ cells for 5 days. Under such conditions, 10% of T cells underwent at least one division cycle at day 7. When CD2– cells were pre-incubated for 2 days in the presence of allogeneic irradiated PBMCs and then cultured for 5 days with CSFE-labeled autologous CD2+ cells, the percentage of divided T cells increased to reach 23.42 ± 7.56%. When allogeneic irradiated PBMCs were replaced by allogeneic Apo-cells, the percentage of divided T cells dropped to 6.32 ± 1.56% (Figure 4E). Altogether, these results demonstrate the low allogeneic priming capacity of human Apo-cells both in the direct and indirect presentation pathways.

### Apo-Cells as a Tool to Modulate Allogeneic Immune Response

**Figure 5:**
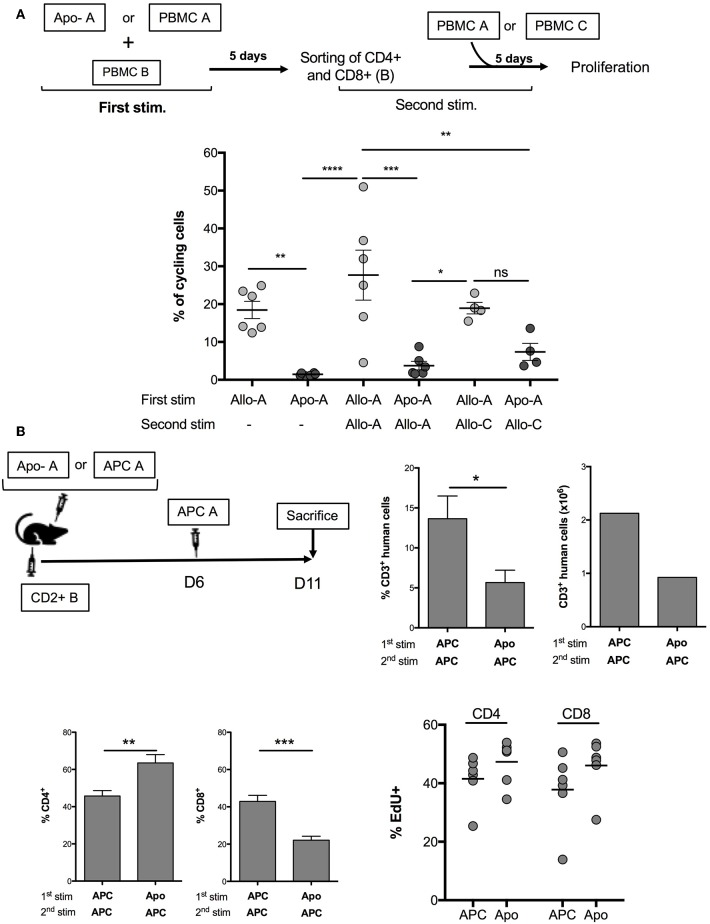
Apo-cells as a tool to modulate allogeneic immune response. **(A)** Experimental scheme. PBMCs were cultured for 5 days in the presence of either allogeneic Apo-cells or irradiated PBMCs. At day 5, T cells were sorted out by flow cytometry, CFSE-labeled, and cultured in the presence of allogeneic irradiated PBMCs from the same donor or from third-party donor. Cumulative individual and mean ± SEM data from six independent experiments. Statistics: one-way ANOVA with *post-hoc* (*p < 0.05, **p < 0.01, ***p < 0.001, ****p < 0.0001). **(B)** *In vivo*, co-injection in NOD/SCID/γC (NSG) mice of either allogeneic CD2– cells (APC) or allogeneic Apo-cells with CD2+ cells (containing mainly T and NK cells). Five days later, mice received an additional dose of allogeneic CD2– (APC) cells from the same source used for the first stimulation. 5-Ethynyl-2′-deoxyuridine (EdU) was injected at days 10 and 11 (2 h before sacrifice). Top right, percentages and numbers of human CD3+ cells at day 11 in the spleen of mice (CD3+ in huCD45+). Bottom left, CD4 and CD8 percentages in CD3+ cells, and percentages of EdU+ in CD4 and CD8 cells. Data represent two independent experiments, each with three mice per group. Statistics: unpaired Student *t*-test (*p < 0.05, **p < 0.01, ***p < 0.001).

Our aim was to assess the possibility of using allogeneic Apo-cells as a strategy of tolerance induction in transplantation. *In vitro*, we developed a procedure consisting of alloantigen sensitization followed by a step of recall with the same antigen, thus simulating organ transplantation consecutive to Apo-cell administration. For such, PBMCs were cultured for 5 days in the presence of either allogeneic Apo-cells or irradiated PBMCs. Then, T cells were sorted out using flow cytometry, CFSE-labeled, and cultured in the presence of allogeneic irradiated PBMCs from the same donor (Figure 5A, top). After 5 days of culture, 18.47 ± 2.28% of T cells cultured with allogeneic PBMCs had divided at least once. After recall antigen stimulation, divided cells represented 27.68 ± 6.6% of sorted out T cells. When the first stimulation was performed with allogeneic Apo-cells, we could not detect any division, as we saw in Figure 3A. After priming PBMCs with Apo-cells and having sorted out T cells cultured in the presence of irradiated allogeneic PBMCs, only 3.75 ± 1.14% of T cells divided, a percentage statistically inferior to that observed in the control group (27.68 ± 6.6%), and even lower than that observed with PBMCs after the first simulation with allogeneic irradiated PBMCs (Figure 5A). Upon culturing sorted-out T cells in the presence of third-party irradiated PBMCs, we detected a slight but not statistically significant difference from the Apo-A/Allo-A group, suggesting that the mechanism of suppression exerted by Apo-cells was partly Ag-specific. We then worked to reproduce this observation *in vivo* in immune-compromised NSG mice that sustain massive proliferation of human T cells following xeno-GvHD (21–23). Precisely, we wanted to investigate whether Apo-cells could modify human T-cell expansion *in vivo*. The first step of sensitization consisted of co-injecting NSG mice with either allogeneic CD2– cells or allogeneic Apo-cells with CD2+ cells (containing mainly T and NK cells). Five days later, mice received a second dose of allogeneic CD2– cells from the same source used for the first stimulation (Figure 5B, top). By then, spleen cells of NSG mice were collected and showed two main features. First, the percentages of CD3+ cells collected from mice sensitized with allogeneic Apo-cells were dramatically lower than those from mice sensitized with CD2– cells, in consistency with the reduced number of CD3+ cells. We reproduced the same experiment starting with Apo-cells or APCs autologous to CD2+. When the second *in vivo* stimulation was performed with allogeneic APCs, we observed a strong decrease in the percentage and number of CD3+ cells, similar to that obtained when starting with allogeneic cells (Supplemental Figure 2). Second, the CD4/CD8 cell ratio was also dramatically modified to the benefit of CD4+ cells in mice sensitized with Apo-cells as compared with CD2– cell-sensitized mice. The increase in CD4+ T cell percentage was not related to Treg increase (data not shown). We also compared the rate of T-cell division in relationship with the strategy of sensitization, and for such, mice were treated with EdU from day 10 to day 11. We could not detect any statistical difference between CD4 and CD8 T-cell divisions. The slight increase in EdU incorporation observed in mice initially stimulated by Apo-cells could be attributed to the increased lymphopenia-induced proliferation, which had resulted from the reduction in numbers of T cells detected in the spleen of this group of mice (Figure 5B, down).

## Discussion

Inducing donor-specific immunosuppression or long-term tolerance in transplantation has been a challenging hypothesis for several decades fed by clinical observations in transplanted patients combined with widened knowledge on the immune system, and particularly the immunosuppressive role of Apo-cells. A strong rationale came first from kidney-transplanted patients who used to be poly-transfused in order to treat their chronic renal dysfunction-induced anemia. Some of them seemed to have fewer rejection manifestations, while others developed enhanced anti-HLA antibodies with deleterious effects on the outcome of the graft. Such observations have remained unexplained both clinically and in terms of the potentially involved immune mechanisms. The emergence of living donor transplantation incited researchers to develop donor-specific transfusion, which may improve long-term graft survival through specific education of the recipient's cellular immune system toward donor antigens (24). However, solid clinical evidence demonstrated by a randomized clinical trial is still missing, and overall, the immunological mechanisms supporting a tolerance-gaining effect via donor-specific transfusions are still misunderstood. The more recent understanding of the immune modulatory effect of Apo-cells, present in transfusion cellular products, could enlighten our comprehension of a pre-transplant transfusion benefit. Particularly, it could help to develop specific tolerance induction strategies in solid organ transplantation. There is a fairly abundant literature demonstrating the immunosuppressive effect of Apo-cells and the possibility of using them to induce tolerance in transplantation. Most of these studies were conducted on animal models (13, 18, 25–30). However, the possibility of generating reproducible and clinically compatible apoptotic cells from blood samples in humans has not been described yet, and very little is known about the immune-modulatory properties of human apoptotic cells (10, 11, 31).

In this study, we first focused on generating and characterizing human Apo-cells by developing a protocol compatible with clinical practice. For this purpose, we started with peripheral blood cells incubated with 8-MOP and exposed to UV-A. This first step allowed us to define stable and reproducible conditions to produce a cellular base of T and monocyte cells, rich in apoptotic cells and poor in necrotic cells. These cells produce IL-10 and TGF-β, express caspase-3 and Fas, and have an apoptotic immunosuppressive phenotype. Interestingly, the cellular content in different T, B, NK, and myeloid cells was not modified, whereas HLA-DR expression was strongly reduced on macrophages, DCs, and B cells, similar to the expression of co-stimulatory CD86 molecule on DCs and B cells. This decreased HLA and CD86 expression could explain the reduced T-cell activation capacity by Apo-cells we observed. The following step, we tried to test the immunosuppressive properties of Apo-cells and to identify the underlying mechanisms. During the rejection process, the allogeneic immune response is initiated primarily by a direct presentation of the donor antigens, hence the importance of demonstrating the suppressive properties of Apo-cells in the direct pathway. Culturing PBMCs in the presence of allogeneic Apo-cells resulted in a strong reduction of responding cells as compared with irradiated PBMCs collected from the same donor of Apo-cells. Not only was the percentage of responding cell proliferation dramatically reduced but also the percentage of activation/memory markers expressed by T cells. In addition, the concentrations of inflammatory cytokines such as IFNγ, IL-6, or TNF-α were strongly reduced.

In transplantation, antigen presentation could also result from donor alloantigen processing by recipient APCs and their presentation to autologous responding T cells. This process is called indirect presentation pathway. We observed that allogeneic Apo-cells had a weak ability to activate APCs (in our model, CD2– cells), as reflected by the reduced HLA-DR expression, and showed a decrease in intensity of expression of co-stimulatory molecules and a decrease in IL-6 and TNF-α production. Finally, we aimed at testing the possibility for Apo-cells to suppress alloreactive immune response generated by an indirect presentation pathway both *in vitro* and *in vivo*. For such, after a first phase of 5-day *in vitro* incubation of PBMCs with allogeneic Apo-cells, CD4 and CD8 cells were sorted out and directly restimulated with allogeneic PBMCs from the same donor of Apo-cells. The percentage of cycling T cells was close to zero even after recall stimulation. Importantly, the application of such a tolerance induction protocol by Apo-cells *in vivo* (in NSG mice) helped us observe a decreased T-cell expansion *in vivo*.

In summary, this work allowed us to define an Apo-cell generation protocol that is compatible with clinical practice. The obtained Apo-cells and their immunosuppressive properties were phenotypically and functionally characterized *in vitro* and *in vivo*. We demonstrate for the first time that Apo-cells can inhibit allogeneic immune response that follows both direct and indirect alloantigen presentation. These results brought the rationale for the use of Apo-cells in tolerance induction protocol in organ transplantation. They also provide solid evidence for the registration of an early-phase clinical trial.

## Data Availability Statement

The datasets generated for this study are available on request to the corresponding author.

## Ethics Statement

Ethical review and approval was not required for the study on human participants in accordance with the local legislation and institutional requirements. The patients/participants provided their written informed consent to participate in this study. The animal study was reviewed and approved by Ethics Committee for Animal Experimentation Charles Darwin (Ce5/2012/025).

## Author Contributions

CP, PG, HR, FP, PL, and JC designed the experiment. CP, TS, AB-F, AT, AB, and CG performed the experiments. CP, TS, BB, MM, and GM analyzed the data. CP, JC, and PG wrote the manuscript.

### Conflict of Interest

The authors declare that the research was conducted in the absence of any commercial or financial relationships that could be construed as a potential conflict of interest.
