# Supplementary figures and images for: Human Apoptotic Cells, Generated by Extracorporeal Photopheresis, Modulate Allogeneic Immune Response
Source: Front Immunol. 2019 Dec 18;10:2908. doi: 10.3389/fimmu.2019.02908 (PMC6930166; doi:10.3389/fimmu.2019.02908)

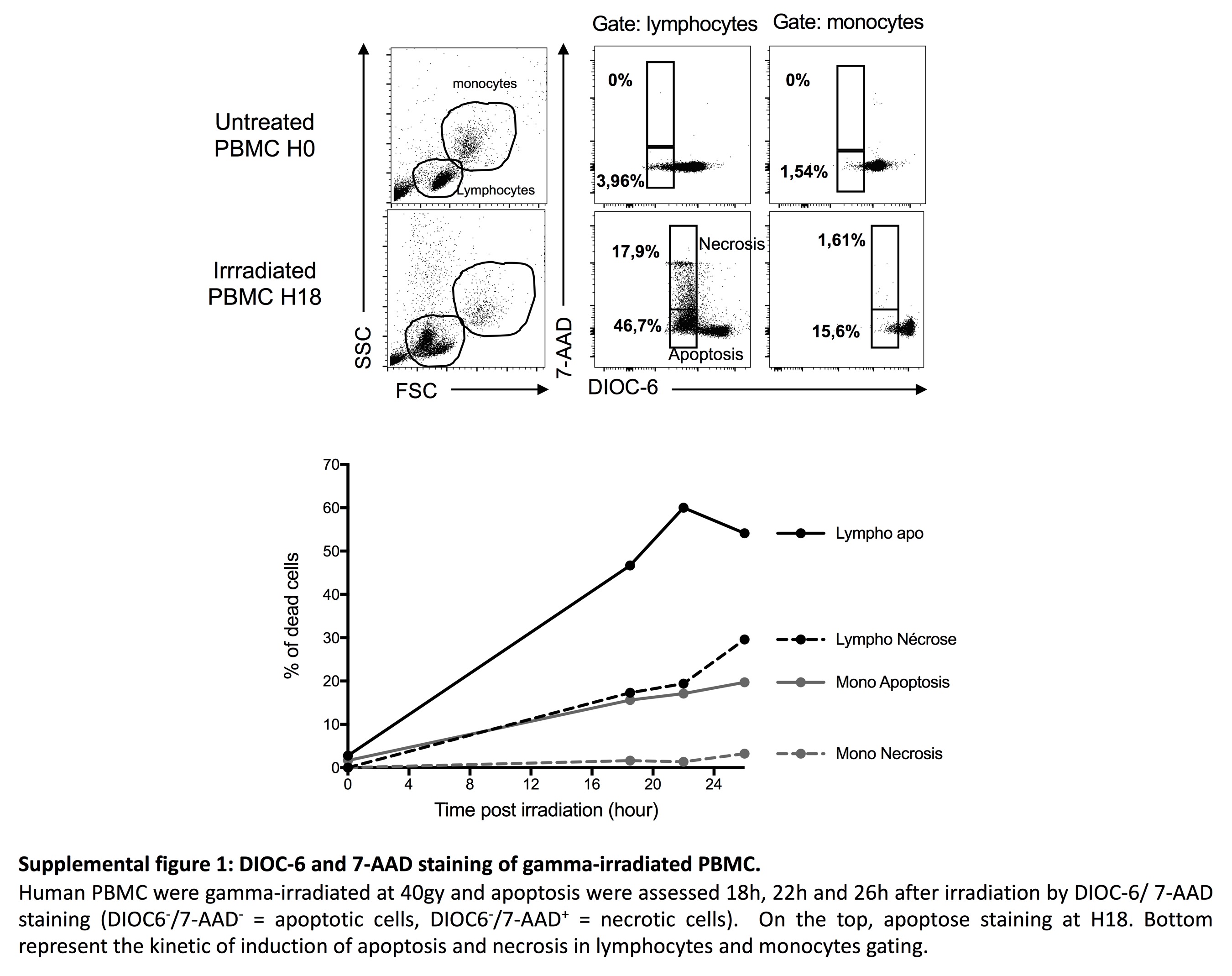

Supplement: Supplementary file 1 [file Image_1.jpg]

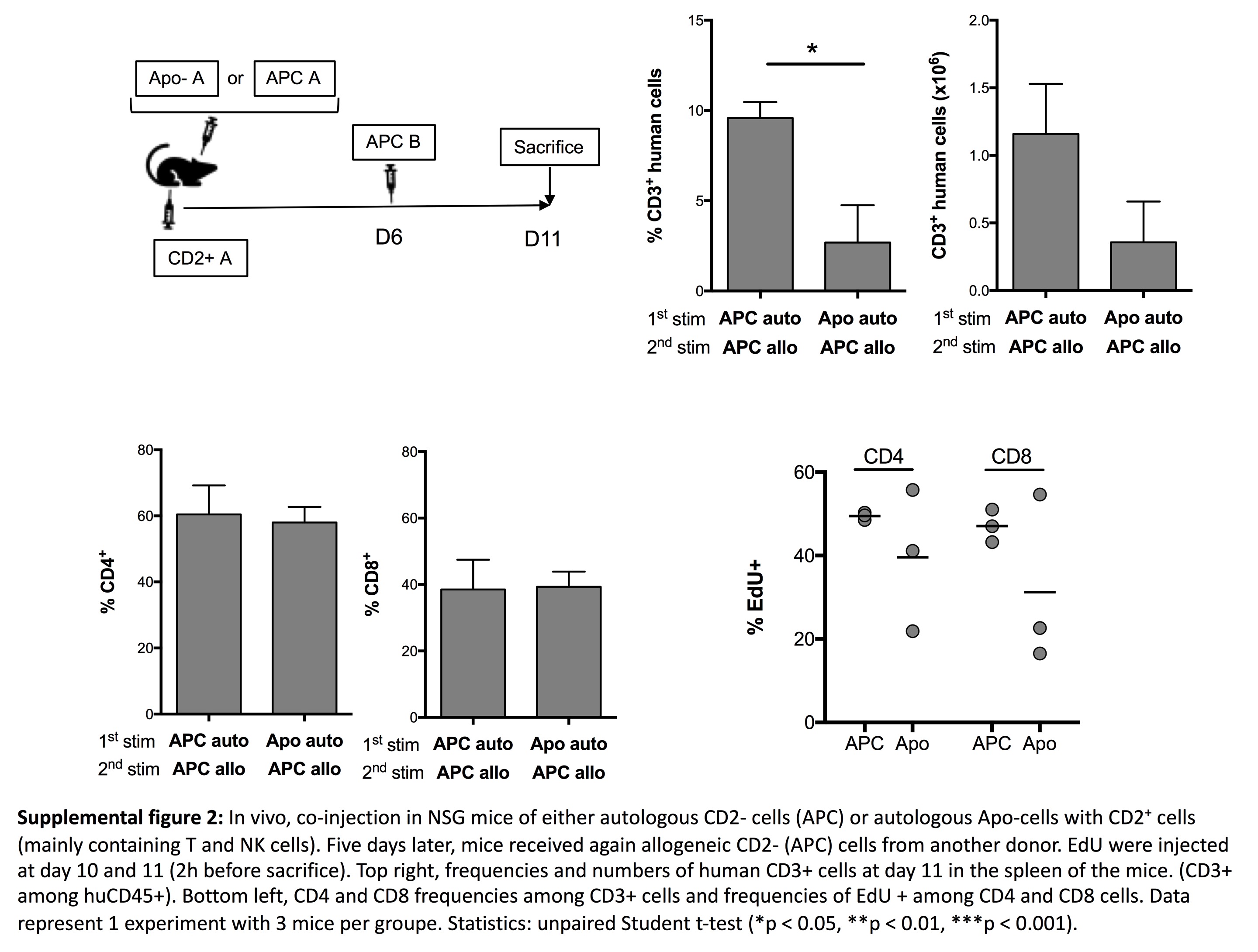

Supplement: Supplementary file 2 [file Image_2.jpg]
